# A Serious Game to Increase Healthy Food Consumption in Overweight or Obese Adults: Randomized Controlled Trial

**DOI:** 10.2196/games.5708

**Published:** 2016-07-13

**Authors:** Tegan Blackburne, Alexandra Rodriguez, Stuart John Johnstone

**Affiliations:** ^1^ Brain and Behaviour Research Institute School of Psychology University of Wollongong Wollongong Australia

**Keywords:** obesity, inhibition training, event-related potential, eating, mobile apps

## Abstract

**Background:**

Obesity is a growing global issue that is linked to cognitive and psychological deficits.

**Objective:**

This preliminary study investigated the efficacy of training to improve inhibitory control (IC), a process linked to overeating, on consumption and cognitive control factors.

**Methods:**

This study utilized a multisession mobile phone–based intervention to train IC in an overweight and obese population using a randomized waitlist-control design. A combination of self-assessment questionnaires and psychophysiological measures was used to assess the efficacy of the intervention in terms of improved general IC and modified food consumption after training. Attitudes toward food were also assessed to determine their mediating role in food choices. A total of 58 participants (47 female) completed 2 assessment sessions 3 weeks apart, with 2 weeks of intervention training for the training group during this time. The groups did not differ in baseline demographics including age, body mass index, and inhibitory control.

**Results:**

Inhibitory control ability improved across the training sessions, with increases in P3 amplitude implying increased cognitive control over responses. Inhibitory control training was associated with increased healthy and reduced unhealthy food consumption in a taste test and in the week following training, as measured by the Healthy Eating Quiz and the food consumption test. Cognitive restraint was enhanced after training for the training but not the waitlist condition in the Three-Factor Eating Questionnaire, implying that attempts to avoid unhealthy foods in the future will be easier for the training group participants.

**Conclusions:**

Inhibitory control training delivered via a purpose-designed mobile phone app is easy to complete, is convenient, and can increase cognitive restraint and reduce unhealthy food consumption.

**Trial Registration:**

Australian New Zealand Clinical Trials Registry ACTRN12616000263493; http://www.ANZCTR.org.au/ACTRN12616000263493.aspx (Archived by WebCite at http://www.webcitation.org/6ioHjGING)

## Introduction

The prevalence of obesity has reached extreme proportions on a global scale. More than 2.1 billion people—one-third of the world’s adults, and one-fourth of children and adolescents—are classified as overweight or obese [1]. Generally speaking, obesity is attributed to increased calorie intake, unhealthy changes in diet composition, and an increasingly sedentary lifestyle [2,3]. In modern society, high-calorie foods are tasty, affordable, and abundant, making them increasingly difficult to resist [4,5]. Healthy foods include fresh fruits and vegetables, as well as nuts, seeds, and lean meat, whereas unhealthy foods include processed meats, potato chips, and butter [6]. Behavioral interventions such as low-calorie meal plans are frequently implemented, but these approaches are often only effective in the short term. They also assume that individuals have the capacity to change their behavior, which is typically not the case [7,8]. An emphasis on deficits in executive functions (ie, the set of processes that manage, regulate, and control cognition and behavior) associated with excess weight, such as poor inhibitory control (IC), may add substantially to the outcomes of obesity interventions.

Inhibitory control refers to the ability to suppress dominant or automatic responses and allows for self-regulation by constraining thoughts and actions that may interfere with the completion of goal-directed actions [2,3,9]. Individuals with deficits in IC are more likely to be unsuccessful dieters and overweight or obese [4,5,10]. Individuals with poor IC tend to engage in unhealthy food consumption behaviors including overeating, excessive snacking, and binge eating [11]. According to dual-process models of obesity [2], the difference between healthy weight and overweight individuals is not the degree of positive affect they experience toward high-calorie foods but the ability to override automatic response tendencies toward consumption [5,12].

There is increasing evidence that the neural mechanisms underlying executive functions are responsive to training and that general IC may be conceptualized as a “muscle” that can be strengthened with exercise [13,14]. Go/Nogo tasks have been widely utilized to train IC, particularly in the context of reducing alcohol consumption [7,15]. These tasks involve making frequent and rapid responses to “Go” stimuli, while refraining from responding to less frequent “Nogo” stimuli [16]. Repeated pairing of inhibition responses to a stimulus creates an association between that stimulus and the goal of stopping behavior [7]. Undertaking training that attaches a stop goal to unhealthy foods may therefore help individuals with low levels of IC to inhibit unintentional impulses toward them.

Intervention studies indicate that IC training is an effective tool in reducing food intake and consumption of high-calorie foods in individuals with low trait IC [5,17]. These studies have focused on immediate taste tests in the laboratory to measure efficacy, leaving the longer-term effects of the training unexplored. Furthermore, most IC training studies in the food domain utilize only a single session of training, which may not be sufficiently intensive to produce improvements [7,10]. Few studies have used multisession, longer-term interventions. Allom and Mullan [18] utilized a stop-signal IC training, focused on weight loss, and did not include psychophysiological measures. Veling et al [19] used a Go/Nogo task delivered via the Internet in 4 weekly sessions and found that this training facilitated weight loss, but they did not assess other outcome measures. Additionally, some research has shown that delivering health interventions through mobile phones has already been studied, targeting exercise and movement, with mixed results [20,21].

Training tasks that have a food-specific, as opposed to a general, focus (using nonfood stimuli), have been found to yield greater improvements in food consumption behaviors [5,17]. Furthermore, current IC training studies have shown that priming disinhibition toward unhealthy foods can increase unhealthy food consumption after training [7,10], but no study has primed disinhibition toward *healthy* foods to determine if IC training can be used to increase healthy food consumption.

Event-related potentials (ERPs) are derived from the electroencephalogram (EEG) and provide an objective index of the sensory and cognitive processing stages involved in processing a stimulus event. In the context of this study, ERPs allow consideration of the neural correlates of inhibition elicited during Go/Nogo tasks, with the N2 and P3 ERP components showing sensitivity to inhibitory demands. The N2 component typically occurs between 200 and 300 milliseconds after stimulus onset [22] and has been positively correlated with body mass index (BMI) [23]. The P3 component typically occurs between 300 and 500 milliseconds after stimulus presentation [16], with neural sources in close proximity to motor and premotor cortices—thus, it is likely that the P3 reflects a later stage of the inhibitory process [24]. Very few studies have examined the neural correlates of inhibition in the context of obesity and food consumption. It has been reported that individuals in a healthy weight range exhibit more pronounced “Nogo” N2 and P3 components, indicative of heightened IC, toward food stimuli as opposed to general stimuli [25].

This study was a pilot study focused on researching the efficacy of an app-based intervention and its effect on short-term weight-related goals (ACTRN12616000263493). It utilized a randomized waitlist-control design and a novel IC intervention to train inhibition toward unhealthy foods and approach (or “disinhibition”) toward healthy foods in individuals identified as overweight or obese. Multisession IC training was employed, as more intensive exercise of neural inhibitory “muscles” should produce greater improvements in IC. This study also took a novel approach by (1) evaluating food consumption over a 1-week period, rather than immediately after training, (2) priming dominant automatic responses (or “disinhibition”) toward healthy foods to increase healthy food consumption, and (3) combining behavioral and psychophysiological measures. Attitudes toward dieting and food were also measured. It was hypothesized that participants in the training condition would consume less unhealthy and more healthy foods than the waitlist condition in the laboratory consumption test and over the 1-week period after training.

Furthermore, an exploratory hypothesis suggested that participants in the training compared with the waitlist condition would show (1) improved performance (reflected by less inhibition errors and faster reaction times) and (2) enhanced N2 and P3 amplitudes, after training in an untrained, non–food-specific Go/Nogo task. Previous research has focused on assessing the generalization of IC abilities from general to specific, or from general to general stimuli; this research is seeking to assess how this process may occur in the opposite direction. Additionally, in accordance with previous research showing changes in attitudes toward food and dieting after weight-based interventions [26,27], it was hypothesized that there would be an increase in cognitive restraint and a decrease in disinhibition and hunger after training in the training but not the waitlist condition in the Three-Factor Eating Questionnaire (TFEQ). Overall, we expected that the training would increase healthy food consumption, reduce unhealthy eating, and this would be reflected in not only eating behaviors but also psychological and neurological changes.

## Methods

### Participants

**Figure 1:**
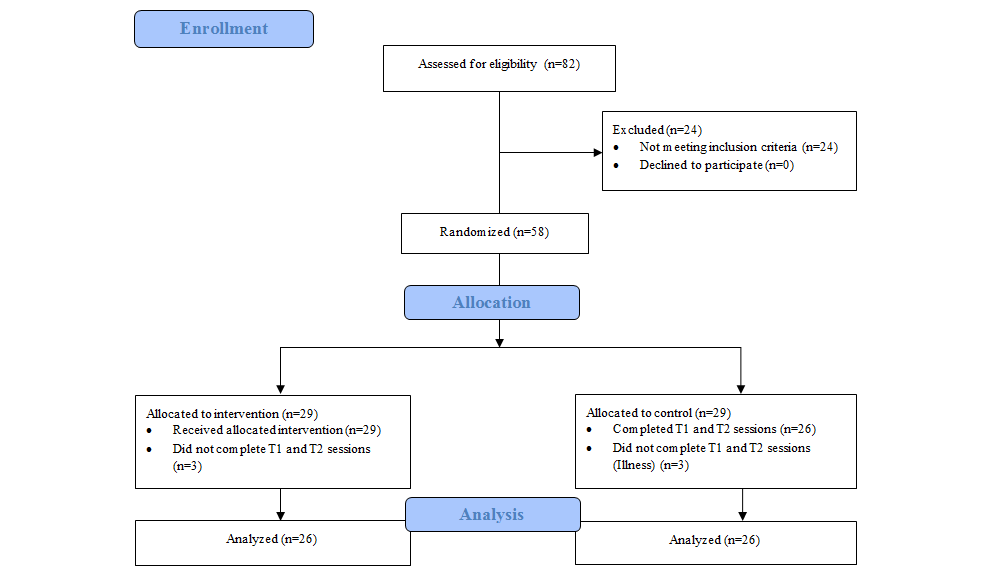
CONSORT (Consolidated Standards of Reporting Trials) Flow diagram. Representation of the progress of participants through the trial. T1: time 1; T2: time 2.

Participants (n=58; females=47, 81%) were recruited from the general population in the Wollongong (Australia) area through flyers distributed in the community. Flyers called for individuals who wanted to improve their eating habits, and the intervention was described broadly as “brain training,” with no specific mention of IC. Participants were required to be older than 13 years (range 19-61, mean 36.48, SD 14.22), have a BMI higher than 25 (mean 29.54, SD 4.05), and possess an iOS device in order to access the training app. Participants were screened for neurological disorders and normal hearing and vision. Participants were excluded if they did not complete both experimental sessions (n=6), leaving a final sample of 52 participants (females=41). Figure 1 shows the CONSORT (Consolidated Standards of Reporting Trials) flow diagram. An a priori power analysis using the G*Power computer program [28] indicated that a total sample of 54 people would be needed to detect moderate effects (*d*=0.5) with 95% power using a *t* test between means with alpha at .05. Baseline differences between the conditions for BMI, age, and number of days between sessions were nonsignificant. Gender ratio was equal across conditions (21 females to 5 males).

### Materials

#### Barratt Impulsiveness Scale

The Barratt Impulsiveness Scale (BIS) is a 30-item questionnaire rated on a 4-point Likert scale and includes 3 subscales: attentional, motor, and nonplanning [29]. It has previously been used to assess the effect of impulsivity on food consumption [30,31]. All participants completed the BIS during their first experimental session to assess potential baseline impulsivity differences between conditions, as this can affect IC [32] and unhealthy food consumption choices [33]. No significant differences were found, and thus the BIS was not included in further analysis.

#### Training Task

**Figure 2:**
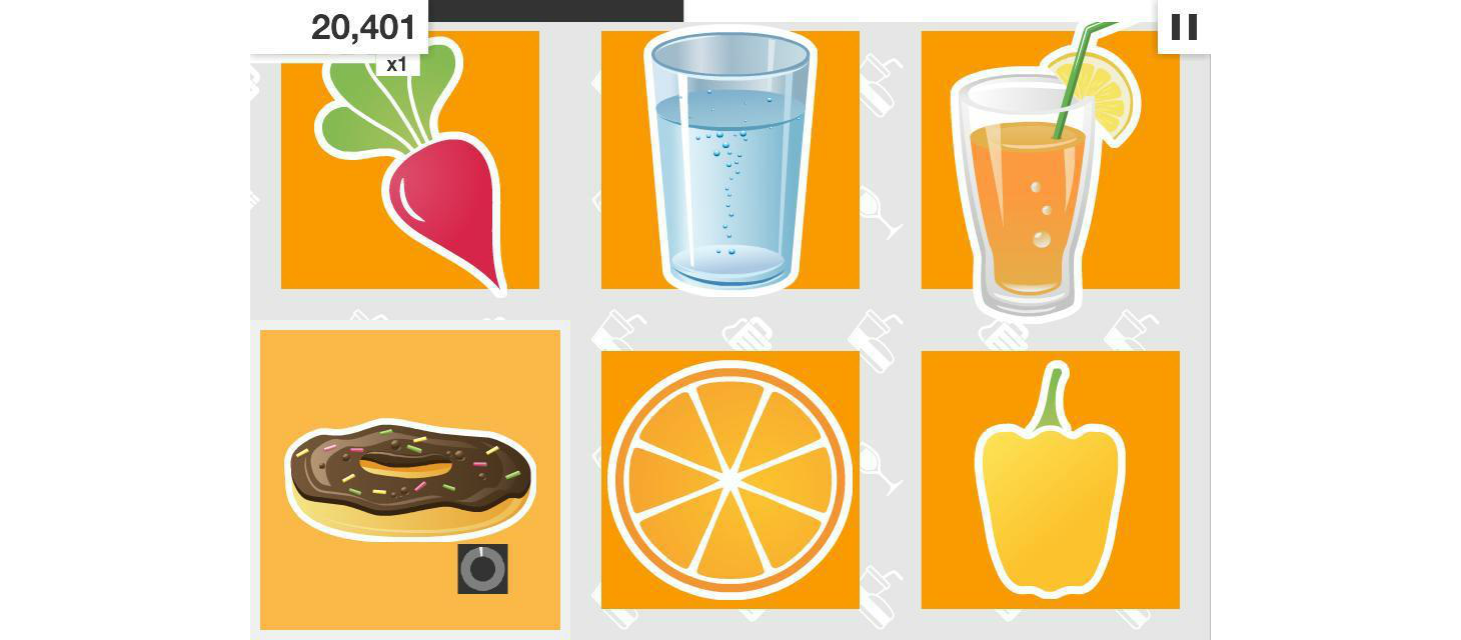
Example of the NoGo app environment with “unhealthy” (doughnut, orange soft drink) and “healthy” (radish, capsicum, water, orange) image categories. This example shows level 6—there are 6 images shown simultaneously, with the active image (requiring a response or not) indicated by the reaction time deadline timer (below doughnut).

The training was delivered via the NoGo iOS app, developed by Neurocog Solutions Pty Ltd, Australia. The NoGo app contains IC training in 3 domains (unhealthy eating, smoking, alcohol consumption)—the training mechanism and parameters were designed by SJ informed by IC training literature. The training task was based on modified Go/Nogo and stop-signal tasks, with each “game” containing (1) Go and Nogo trials in which the image remained the same when the reaction time deadline (RTD) timer appeared next to the image and started to count down and (2) stop trials in which the image could change from healthy to unhealthy after the timer appeared. Participants were instructed to tap the images of healthy food as quickly and accurately as possible before the RTD expired and to refrain from tapping images of unhealthy food. Each game consisted of 30 trials, using stimuli drawn from a pool of food-specific images. For the purpose of creating a clear divide between different food types, the presented stimuli were in 2 categories: healthy (eg, fruits, vegetables) and unhealthy (eg, chips, doughnuts) foods (Figure 2).

Participants were required to play 10 games per day for 14 consecutive days with each game taking approximately 1 minute to complete. The difficulty level of the game increased according to past performance by reducing the RTD and increasing the number of images presented at one time (maximum 12 images). Go stimuli were presented on 70%-90% of occasions, varying randomly each game to ensure individuals were responding genuinely to presented stimuli and were not prepreparing responses based on previous patterns of stimulus presentation.

A log of performance data including reaction time, game level, correct responses, and errors was stored locally and accessed by researchers at the end of the training period. For analysis purposes, and in order to assess potential improvements in NoGo app performance as game play progressed, the training data of each individual participant were split into 3 sessions. Sessions 1, 2, and 3 comprised an equal number of games for each participant (eg, if a participant completed 120 games, session 1 would consist of data collected from games 1 to 40, session 2 would consist of games 41 to 80, and session 3 would consist of games 81 to 120). Reaction time, game level, and correct/incorrect response scores were averaged for each participant across each session to create 3 data points, which were used to assess improvements in game-play performance across all participants. Although participants in the training condition were expected to play a total of 140 games, an arbitrary cutoff of 90 games was selected, reflecting a 65% adherence rate. Participants who failed to play 90 games over the 14-day period were excluded from further analysis.

#### Healthy Eating Quiz

The Healthy Eating Quiz (HEQ) is a food frequency questionnaire containing 70 items relating to the frequency of healthy food consumption over the past 7 days, rated on a Likert scale from “never” to “5 or more times a week.” It includes 8 subgroups: fruits, vegetables, meat proteins and vegetarian proteins, grains, water, dairy, and extras (ie, sauces and spreads). Participants were asked to rate approximately how many servings of items within these food groups they had consumed in the past week. Food frequency questionnaires are widely used to assess eating habits [34,35].

#### Food Consumption Test

The food consumption test (FCT) was used to examine immediate changes in food consumption after the IC training. It was based on the bogus taste tests used in previous research but without the associated element of deception [2,7,10]. Participants were informed that they would receive a refreshment break during the session in which snacks would be offered and that they could eat as little or as much as they liked. Participants were presented with 4 bowls containing snack-sized portions of 2 healthy (156 g of unsalted nuts and 216 g of grapes) and 2 unhealthy food options (60 g of plain potato chips and 161 g of chocolate candies, M&M's). Although nuts and grapes are still high-calorie choices, these foods were chosen as they represent “real-world” choices that participants would be faced with. For example, although grapes are still high in sugar, they are high in natural sugar and are nonprocessed. Thus, they represent a healthier choice than M&M's (which are processed and high in sugar and saturated fat) for individuals when craving a convenient, sweet snack food. Food was presented for 10 minutes while the experimenter cleaned equipment used previously in the experimental session, facing away from participants. After the participant left the laboratory, the food was weighed, consumption was recorded, and the number of calories consumed was calculated. Before completing the FCT, participants were asked covertly (questions were embedded in a larger general health questionnaire) to rate their current levels of hunger and food craving to account for differences in the FCT results between conditions. As no significant differences were found for hunger *F*_1,48_=0.24, *P*=.05 or craving *F*_1,48_=0.00, *P*=.99, these factors were not included as covariates in further analysis.

#### Three-Factor Eating Questionnaire

Participants completed the 51-item TFEQ [36] that contained a combination of true or false questions (eg, “Sometimes when I start eating, I just can’t seem to stop”) and items rated on a 4-point Likert scale (eg, “How frequently do you avoid ‘stocking up’ on tempting foods?” Rated: not at all, slightly, moderately, or almost always). The TFEQ contains 3 subscales (cognitive restraint, disinhibition, and hunger) measuring the ability to resist foods and overcome internal and external food cues. The TFEQ has been widely used in overweight and obese populations [26,27] and is psychometrically valid in obese and nonobese populations [37].

#### Go/Nogo Task

Two auditory Go/Nogo tasks that varied in RTD were used to assess general IC ability. Tones were presented at 1100 Hz and 2000 Hz for 200 milliseconds, with an interstimulus interval (ISI) of either 2500 milliseconds (longer RTD) or 1250 milliseconds (shorter RTD). These specific time intervals were pilot-tested for this study in an adult population. Shorter RTDs increase task demands by inducing rapid responding to Go stimuli making the inhibition of motor responses on infrequent “Nogo” trials more difficult and increase the likelihood of errors [13]. As a result, this study used 2 separate tasks with varying task demands (“longer RTD” and “shorter RTD”) to better assess potential effects of the IC training. Tasks were created in Presentation version 11, and tones were presented at 60 dB binaurally through Sennheiser HD 201 headphones. Participants were required to press a response button on a game controller upon presentation of the frequent “Go” tone (70% of presented stimuli) and to withhold response upon presentation of the infrequent “Nogo” tone. Participants completed 10 practice trials to ensure they understood task instructions and then completed 100 trials, taking approximately 2.5 minutes to complete. Assignment of each tone as Go/Nogo, and the order of tasks, was counterbalanced across sessions and participants. This task was used to assess how the IC training affected general IC ability by using a task involving similar processes but sharing few surface features and different modality (auditory rather than visual) to reduce practice effects and make comparison with the control group more equal.

### Procedure

In the first experimental session (around 90 minutes) participants gave informed consent and then completed the BIS, Go/Nogo tasks, FCT, HEQ, and TFEQ. Participants also completed a passive image-viewing task, not reported here. After completing this session, participants in the training condition were given access to the training app and instructed to play 10 games a day for 14 consecutive days. Participants performed a trial game with the researchers to provide them with the opportunity to ask questions and understand the game format. Participants were then sent reminders to play the game via email on days 1, 7, and 14 to ensure compliance. During the second session, approximately 3 weeks since session 1 and 1 week since the end of the training (average time between sessions was 4.5 weeks but did not differ between conditions), individuals repeated the same procedure, game data were obtained from the training participants, and the waitlist-control condition received access to the training app. Participants were entered into a prize draw to win 1 of 4 A$100 gift vouchers after completing the study, as reimbursement for their time.

#### Event-Related Potential Recording and Quantification

Electroencephalographic data were recorded at 9 sites (F3, Fz, F4, C3, Cz, C4, P3, Pz, and P4) using a 19-site cap and a Nuamps amplifier (Compumedics, Melbourne Australia). Vertical and horizontal electro-oculogram was recorded using electrodes placed above and below the left eye and near the outer canthus of each eye. Linked ears were used as a reference, and an electrode located between Fz, Fp1, and Fp2 acted as a ground electrode, with impedances kept below 5 kΩ. Signals were amplified 19 times with a band-pass down 3 dB at 0.01 and 100 Hz. Raw EEG data were visually inspected and sections of muscle artifact were removed. Data were low-pass filtered down 3 dB at 24 Hz and divided into epochs from −100 milliseconds prestimulus to 900 milliseconds poststimulus. Epochs were excluded if they contained amplitudes outside ±100 µV at any EEG site. The number of accepted epochs for Go and Nogo stimuli were compared between conditions, with no differences evident. ERPs were calculated separately for correct Go and Nogo trials. Grand mean ERPs were visually inspected to identify major peaks. Peak quantification was completed via a computer algorithm, which allows for the automatic identification of the maximum or minimum voltage occurring within a specified latency window. For N2, peak identification at all other sites was locked to the largest negativity at Fz in the 190- to 300-millisecond latency window, whereas the P3 was locked to the largest positivity at Pz in the 300- to 580-millisecond window as per Johnstone et al [38].

#### Statistical Analysis

One-way analysis of variance (ANOVA) was used to assess baseline differences between training and waitlist conditions and to assess NoGo training app performance data. Additionally, time (1, 2) × condition (waitlist, training) mixed design ANOVA was used to assess the FCT, HEQ, and TFEQ, with planned follow-up analysis splitting data by condition and assessing the effect of time. ERP component latency analysis was restricted to the frontal midline location (Fz), as the N2 and P3 components showed a frontal maximum. Go and Nogo ERP data were subject to a condition × time × stimulus (Go, Nogo) × RTD (longer, shorter) mixed factorial ANOVA. ERP amplitude analyses included an additional lateral (left, midline, right) factor. Planned contrasts within the lateral factor compared the right and left hemispheres. Here we focus on interactions between time and condition, and time × condition × laterality for ERP amplitude analysis. Alpha level was set to .05 for all analyses.

## Results

### Baseline Characteristics

Table 1 shows the analysis comparing the waitlist and training groups at session 1. As participants did not differ statistically in any of these variables, they were not included as covariates in future analysis. Both groups had equal numbers of male and female participants (19% male, 11/58).

**Table 1:** Baseline analysis results for training and waitlist conditions.

Demographics		Training		Waitlist		*F* (*df*)	*P* value
		Mean	SD	Mean	SD		
Age, years		35.2	14.1	38.3	14.8	0.56	.46
Body mass index		29.7	4.4	29.2	3.5	0.19	.67
Return time, days		33.5	15.5	31.2	12.5	0.34 (1,46)	.56
**BIS** ^a^ **- subscale**							
	Attentional	17.9	3.4	17.0	2.8	1.03	.32
	Motor	21.6	3.8	22.3	3.7	0.40	.53
	Nonplanning	23.5	3.9	22.6	5.2	0.54	.46

^a^ BIS: Barratt Impulsiveness Scale.

### Training Performance

For participants completing the NoGo training, 1-way repeated measures ANOVA was used to assess changes in reaction time across the 3 training session blocks. The main effect of time was significant (*F*_2,46_=156.80, *P*<.001, partial *η*^2^=.87). Planned contrasts indicated that average reaction time was reduced at session 2 (mean 330.8 milliseconds, SD 26.03) compared with session 1 (mean 401.7 milliseconds, SD 44.7), *F*_1,23_=133.26, *P*<.001, partial *η*^2^=.85, and reduced at session 3 (mean 307.1 milliseconds, SD 33.2) compared with session 2, *F*_1,23_=35.53, *P*<.001, partial *η*^2^=.61.

The main effect of time was also significant for correct Go responses (*F*_2,46_=82.78, *P*<.001, partial *η*^2^=.79). Planned contrasts indicated that session 2 (mean 53.35, SD 5.48) had more correct Go responses than session 1 (mean 48.22, SD 1.65), *F*_1,23_=24.11, *P*<.001, partial *η*^2^=.52, and session 3 (mean 62.46, SD 5.14) had more correct Go responses than session 2, *F*_1,23_=43.37, *P*<.001, partial *η*^2^=.66. The main effect was also significant for correct Nogo responses (*F*_2,46_=93.07, *P*<.001, partial *η*^2^= .79). There were more correct Nogo responses in session 2 (mean 27.83, SD 9.51) than session 1 (mean 17.57, SD 3.85), *F*_1,23_=53.54, *P*<.001, partial *η*^2^=.69, and more correct Nogo responses in session 3 (mean 42.22, SD 14.62) than session 2, *F*_1,23_=80.19, *P*<.001, partial *η*^2^=.77. Overall, these results indicate that participants in the training condition showed improved IC performance with increased training.

### Healthy Eating Quiz

The total HEQ score showed a time x condition interaction (*F*_1,48_=9.87, *P*=.003, partial *η*^2^=.17). Simple effects analysis showed a significant effect of time for the training condition (*F*_1,25_=5.21, *P*=.03, partial *η*^2^=.03), with an increase in healthy food consumption from time 1 (mean 36.96, SD 8.45) to time 2 (mean 42.42, SD 9.98). The waitlist condition also showed a significant time effect (*F*_1,25_=7.88, *P*=.01, partial *η*^2^=.01), with a decrease in healthy food consumption between time 1 (mean 39.58, SD 9.68) and time 2 (mean 35.88, SD 11.24).

### Food Consumption Test

Calorie consumption was calculated for grapes, mixed unsalted nuts, M&M's, and plain potato chips separately. Then, the “healthier” foods (nuts and grapes) and “less healthy” foods (chips and M&M's) were added together to create 2 food groups for analysis. The condition × food × time ANOVA was significant (*F*_1,48_=8.88, *P*=.005, partial *η*^2^= .18). Follow-up 2-way ANOVA for each condition separately showed no significant interaction for the waitlist condition but a significant food × time interaction for training condition (*F*_1,19_=8.81, *P*=.008 partial *η*^2^= .32). This showed an increase in calorie consumption of healthier foods at time 2 (mean 63.17, SD 19.08) compared with time 1 (mean 31.59, SD 9.47) and a reduction in total calorie consumption of less healthy foods from time 1 (mean 234.68, SD 74.40) to time 2 (mean 76.50, SD 25.15).

### Three-Factor Eating Questionnaire

The disinhibition subscale did not show a time x condition interaction. A significant interaction was found for the hunger subscale (*F*_1,50_=4.07, *P*=.05, partial *η*^2^=.08). Simple effects analysis showed no time effect for the training condition, whereas the waitlist condition showed a significant time effect, *F*_1,25_=10.74, *P*=.003, partial *η*^2^=.30, with an increase between time 1 (mean 5.58, SD 3.46) and time 2 (mean 6.88, SD 3.56). The cognitive restraint subscale also showed a significant time x condition interaction (*F*_1,50_=6.02, *P*=.02, partial *η*^2^=.11). Simple effects analysis indicated no significant time effect for the waitlist condition, but a significant effect was present for the training condition, *F*_1,25_=6.01, *P*=.02, partial *η*^2^=.19, with an increase from time 1 (mean 9.27, SD 3.38) to time 2 (mean 11.5, SD 4.25).

### Go/Nogo Task Performance

For the shorter RTD Go/Nogo task, the time x condition interaction was not significant for correct Go responses or reaction time, or Nogo errors. No significant interactions were found for the same measures on the longer RTD Go/Nogo task.

### Event-Related Potential Components

**Figure 3:**
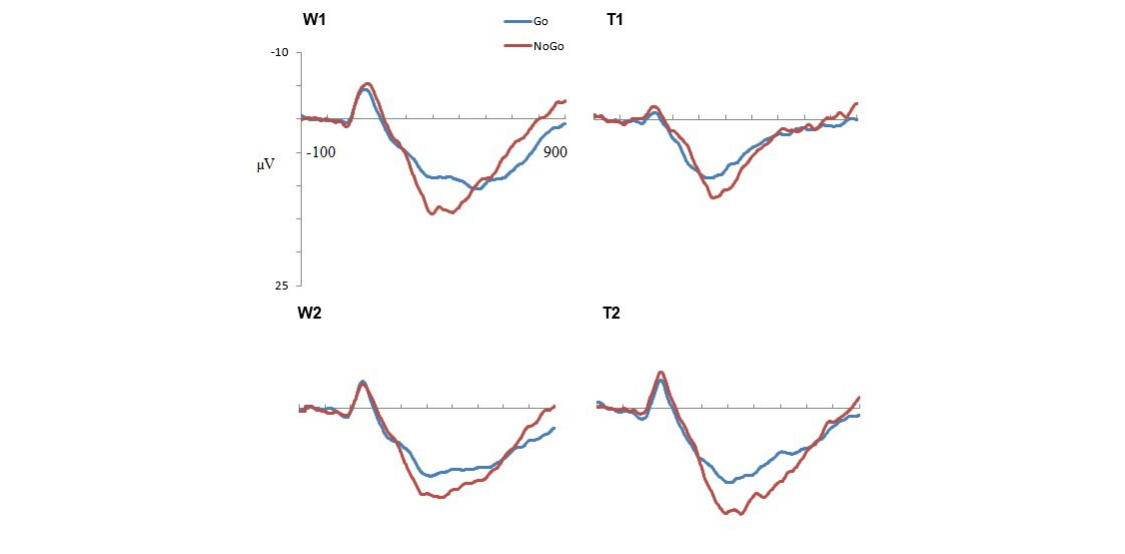
Grand mean event-related potentials at Fz for Go and Nogo stimuli in the longer reaction time deadline task. W1: waitlist time 1; W2: waitlist time 2; T1: = training time 1; T2: training time 2.

**Figure 4:**
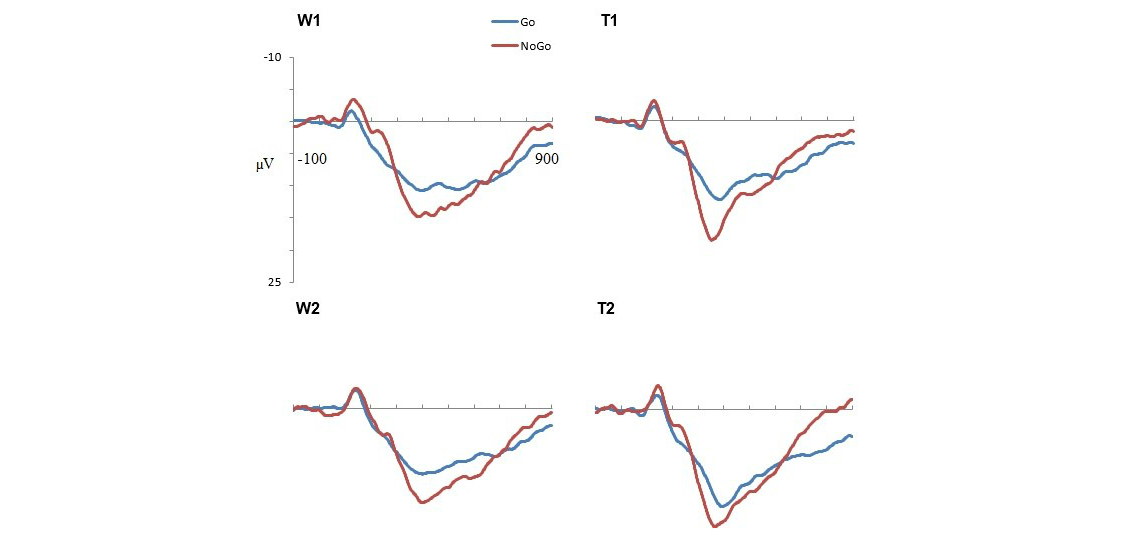
Grand mean event-related potentials at Fz for Go and Nogo stimuli in the shorter reaction time deadline task. W1: waitlist time 1; W2: waitlist time 2; T1: training time 1; T2: training time 2.

**Figure 5:**
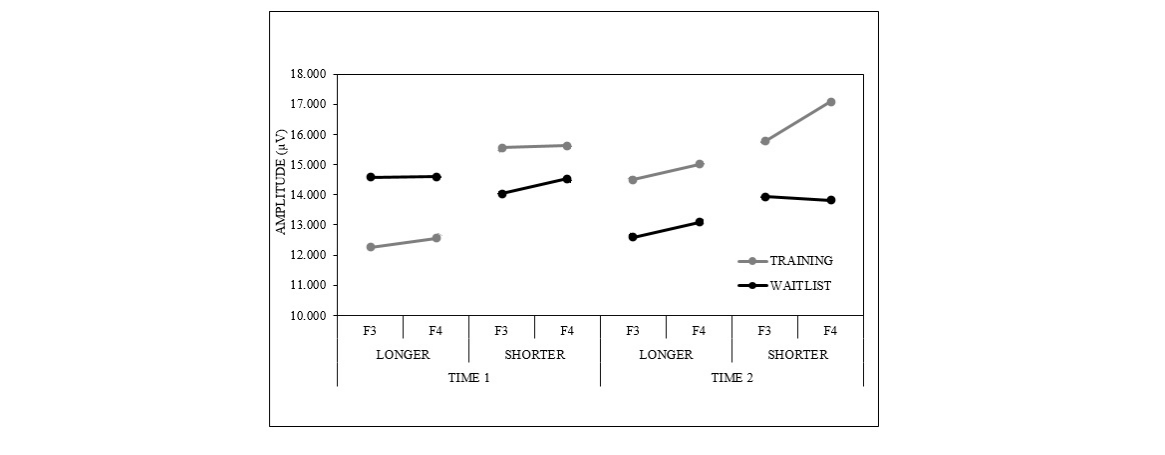
P3 amplitude changes in the Go/Nogo tasks. P3 amplitude at sites F3 and F4 for the Go/Nogo tasks, with each reaction time deadline and time shown separately.

Grand mean ERPs to Go and Nogo stimuli for each condition and time are presented separately for the longer RTD (Figure 3) and shorter RTD (Figure 4) tasks. For the latency analysis, no significant interactions were found for either component. For the amplitude analysis, there were no significant relevant interactions for the N2 component. A significant condition × time × laterality × RTD interaction was found for P3 (*F*_1,46_=8.49, *P*=.005, partial *η*^2^=.16; Figure 5). The waitlist condition showed a reduction (largest in the left frontal region) in P3 amplitude at time 2 compared with time 1 for the longer ISI task and a very minor reduction (largest in the right frontal region) at time 2 compared with time 1 for the shorter ISI task. The training condition showed a very different pattern, with a substantial increase (of similar magnitude in both frontal hemispheres) in P3 amplitude at time 2 compared with time 1 for the longer ISI task and a smaller increase (mainly in the right frontal region) at time 2 compared with time 1 for the shorter ISI task.

### Results Summary

The key significant effects for both groups are presented in Table 2, showing mean changes between time 1 and time 2.

**Table 2:** Mean scores for each outcome; only statistically significant results are shown.

Variable	Participant group			
	Training		Waitlist	
	Baseline	Time 2	Baseline	Time 2
Healthy Eating Quiz	36.96	42.42	39.58	35.88
FCT^a^: healthy food	63.17	31.59	-	-
FCT: unhealthy food	234.68	76.50	-	-
TFEQ^b^: hunger	-	-	5.58	6.88
TFEQ: cognitive restraint	9.27	11.50	-	-

^a^ FCT: food consumption test.

^b^ TFEQ: Three-Factor Eating Questionnaire.

## Discussion

### Principal Findings

This pilot study used a randomized waitlist-control design to examine the efficacy of food-specific IC training in overweight and obese individuals. Traditional measures were used to assess food consumption (HEQ, FCT) and attitudes toward food and dieting (TFEQ). As a measure of general IC ability, Go/Nogo performance data and ERPs were also assessed. Key findings included increased healthy food consumption outside laboratory environments, decreased unhealthy food consumption during testing, and an increase in scores on the cognitive restraint scale for the training group. Condition-specific changes in the P3 ERP component indicated that the long-term, food-specific, IC training had a generalizable effect on IC-related processing, at least in the context of this preliminary research.

This study was novel and exploratory in its linking of healthy foods with approach responses in the context of an intensive multisession training regimen. As hypothesized, a reduction in immediate unhealthy food consumption and an increase in healthy food consumption (both in the laboratory and 1 week after training) was found for participants who completed the training task, supporting previous research [5,10,17,18]. Increases in the HEQ score indicated that individuals reported an increase in healthy food consumption in the week after using the intervention, eating more fruits, vegetables, and protein or drinking more water. This provides encouraging evidence that IC training may be effective in priming approach behaviors toward healthy food, as well as strengthening resistance toward unhealthy foods. Furthermore, the results provide some evidence that these changes may have translated to the participant’s day-to-day life. However, a longer-term unhealthy food consumption measure was not included in this initial research and will be necessary in the future to determine whether decreases in unhealthy food consumption are maintained.

To complement the food consumption data, attitudes toward food were also measured. Participants in the training condition showed increased cognitive restraint, a construct related to self-regulation of food intake on the TFEQ scale. Increased cognitive restraint has been linked to weight loss in dieters and improvements in avoiding unwanted food consumption [26]. Bryant and colleagues [27] showed that a weight loss intervention developed cognitive restraint in some participants, and this resulted in increased weight loss for these participants. Although our study did not monitor weight loss, this increased ability to control calorie intake may assist individuals to achieve weight loss goals, especially as it can easily be used alongside diets such as meal plans [2].

Our study incorporated psychophysiological methods to measure the neural correlates of IC training. Contrary to prediction, N2 amplitude did not increase in the training condition as a result of IC training. This indicates that the IC training did not have a broad effect on the process reflected by the N2 component at either difficulty level. This may be due to the N2 being more closely related to conflict monitoring (a process not targeted by the IC training), as opposed to IC [39,40].

Training effects were present for the P3 ERP component, with amplitude increases after training seen in the training but not the waitlist conditions. This effect could be described as “global” as it did not differ based on the stimulus type (ie, Go, Nogo) and was consistent across the longer and shorter RTD Go/Nogo tasks. However, the nature of the condition effect depended on task difficulty; the shorter ISI task places higher demands on IC as a more speeded response is required to Go stimuli and could be considered more difficult. In the easier Go/Nogo task, the training condition showed a substantial increase (of similar magnitude in left and right frontal regions) in P3 amplitude at time 2, whereas the waitlist condition showed a substantial reduction (largest in the left frontal region). In the more difficult Go/Nogo task, the training condition showed a minor increase in P3 amplitude (largest in the right frontal region), whereas the waitlist condition showed a minor reduction (largest in the right frontal region). In both tasks, rapid responding was induced and thus inhibitory “activation” was required to inhibit automatic motor responses [41]. Given the established relationship between the P3 ERP component and cancellation of a planned response [42,43], these training effects for P3 are consistent with improved IC abilities, with the degree of improvement more evident in the easier of the 2 tasks.

### Limitations

The results reported here should be considered in light of several limitations, primarily because of the limited nature of this preliminary study. Although all attempts were made to recruit adolescent participants, the final sample only included participants older than 18 years, preventing analysis across different age groups. Further research investigating the ideal age to administer IC training is warranted. Adolescents may experience greater benefits from the training, as IC abilities are known to continue developing into adolescence [44]. Other variables that affect food consumption, such as beverage intake [45], mental illness comorbidities [46], and menstrual cycles for women [47], were not assessed and may have affected the outcomes of this study. Race and socioeconomic status were also not recorded, although these can affect access to food and obesity rates [48-50]. As the majority of participants were female, our results cannot be generalized across sex. Females and males have been shown to consume unhealthy food at different rates, and for different reasons, particularly when snacking [51].

Additionally, the study duration was relatively short and did not use weight loss as an outcome measure. A longer-term study that assesses weight loss and maintenance would be beneficial, as previous research has found mixed results for changes in BMI at follow-up [18,19] and additional research is needed to determine how IC training can influence health behaviors. An additional limitation is related to the stimuli used in the NoGo training app versus the foods presented in the FCT. Although grapes were featured in both the training task and the FCT, M&M's, potato chips, and nuts were not. Increases in the consumption of grapes may therefore potentially be attributed to grapes being specifically featured in the training task. Future versions of the FCT should utilize either foods that are all featured in the training task or none at all to ascertain if training on specific stimuli can transfer to general stimuli.

Most importantly, this study has focused on self-report measures, which are inherently biased and vulnerable to demand effects. Although the waitlist-control design helps to reduce the effects of random error, the nonblind randomization of participants means that individuals who know they are in a waitlist versus training group may not respond as they normally would. This may explain why the participants in the waitlist condition ate significantly less healthy food in the HEQ at time 2 and may explain the increase in the disinhibition subscale of the TFEQ. This subscale is linked to making poorer health food choices [52], which may be the result of the control group allowing themselves to “give in” to their food cravings, knowing that they will be receiving a healthy eating intervention after the experimental period. In the same way, those in the training group could easily deduce that experimenters expected them to eat more healthy foods after the training and may have been acting in accordance with this expectation, not due to the training itself. Although utilizing an active control condition (eg, having participants read information about healthy eating choices) is a potential way to reduce demand effects, nonblind assignment to active control groups may still reveal bias results, especially for gaming-based interventions [53].

Future research must be especially careful in controlling for the expectations and beliefs of both the training and waitlist groups or measure these expectations to examine potential differences that may confound results. Further studies therefore need to consider a way of incorporating a more active control group, which will help make the purpose of the intervention less apparent to participants, reducing bias effects. Alternative food consumption measures, such as food diaries or dietary interviews, would also improve the accuracy of these data, although this was beyond the scope of this study. A larger number of participants and an equal number of male and female participants would improve statistical power.

### Conclusions

This study, while preliminary, replicated and extended previous food-related IC training research, by priming both inhibitory responses to unhealthy food and approach responses toward healthy foods. Inhibitory control training was delivered through a purpose-designed, mobile phone app that allowed participants to complete the training at a time and place convenient to them. Further research is required to assess if the observed changes transfer to longer-term real-life contexts, such as weight loss. As it is easy to complete and cheap to obtain, IC training is a promising alternative or addition to existing obesity interventions.

## Abbreviations

ANOVA: analysis of variance
BIS: Barratt Impulsiveness Scale
BMI: body mass index
EEG: electroencephalogram
ERP: event-related potential
FCT: food consumption test
HEQ: Healthy Eating Quiz
IC: inhibitory control
ISI: interstimulus interval
RTD: reaction time deadline
TFEQ: Three-Factor Eating Questionnaire
